# Fecal microbiota transplantation versus glucocorticoids for the induction of remission in mild to moderate ulcerative colitis

**DOI:** 10.1186/s12967-022-03569-3

**Published:** 2022-08-12

**Authors:** Chunlan Huang, Zehua Huang, Ling Ding, Yang Fu, Junjie Fan, Qixiang Mei, Lihong Lou, Jingjing Wang, Nuoming Yin, Yingying Lu, Sanwei Guo, Yue Zeng

**Affiliations:** 1 Department of Gastroenterology, Shanghai General Hospital, Shanghai Jiao Tong University School of Medicine, No. 650 Xinsongjiang Road, Songjiang District, Shanghai, China; 2 Shanghai Key Laboratory of Pancreatic Diseases, Shanghai Jiao Tong University School of Medicine, Shanghai, China; 3 Department of Infectious Diseases, Central People’s Hospital of Zhanjiang, Zhanjiang, Guangdong Province China; 4 International Medical Care Center, Shanghai General Hospital, Shanghai Jiao Tong University School of Medicine, Shanghai, China; 5 Department of Gastroenterology, Jiading Branch of Shanghai General Hospital, Shanghai Jiao Tong University School of Medicine, Shanghai, China; 6 Department of Urology, Shanghai Tenth People’s Hospital, Tongji University, No. 301, Middle Yanchang Road, Jingan District, Shanghai, China

**Keywords:** Ulcerative colitis, Fecal microbiota transplantation, Glucocorticoid, Serum cytokines

## Abstract

**Objective:**

To compare efficacy and safety of fecal microbiota transplantation (FMT) with glucocorticoid as induction therapy in ulcerative colitis (UC).

**Methods:**

The patients with active mild to moderate UC were recruited into the single-center, prospective cohort study. The patients were treated with either FMT (FMT group) or glucocorticoids (GCs group). Patients received FMT administration for 3 days. The primary outcome was clinical and endoscopic remission at week 12. Inflammatory parameters were assessed by routine blood tests. Safety was assessed by adverse events recorded. The serum levels of TNF-α, IFN-γ, IL-1β, IL-4, IL-5, IL-6, IL-10 IL-8, IL-12p70, IL-13, IL-17A and IL-23 following FMT were measured by Luminex multiplex assay.

**Results:**

Of the 122 patients, 62 patients were treated with FMT and 60 with glucocorticoids. 34 patients in FMT group (54.8%) and 29 in GCs group (48.3%) reached the primary outcome (*p* = 0.30). The incidence of adverse events in GCs group (35/60, 58.3%) was significantly higher than that in FMT group (14/62, 22.6%) and two serious adverse events were observed following GCs. Patients in FMT group were stratified into responders (RE) and non-responders (NR) groups. The level of TNF-α and IL-6 decreased significantly in RE group, while IL-10 decreased significantly in NR group.

**Conclusion:**

FMT therapy was as effective as glucocorticoids to induce remission in active mild to moderate UC, accompanied by fewer adverse events. The modification of serum TNF-α, IL-6 and IL-10 might be related to the efficacy of FMT in UC.

*Trial registration* This study was registered with ClinicalTrials.gov (NCT02435160). Registered on 6 April, 2015. https://clinicaltrials.gov/ct2/results?cond=&term=NCT02435160&cntry=&state=&city=&dist=

## Introduction

The ulcerative colitis (UC) is the non-specific chronic bowel inflammation disease with potential systemic complications. Population-based studies have regarded UC as a world-wide public health challenge with an accelerating incidence in the newly industrialised regions such as Asia [1, 2].

UC is classified into mild, moderate and severe disease according to the severity [3]. Mild to moderate severity is defined as less than 6 stools per day with or without blood may with mild anemia or low-grade fevers with no signs of severe systemic toxicity. Early treatment and remission in UC can alter the disease course and prevent long-term complications [4]. Therefore, great importance should be attached to the induction of remission in mild to moderate UC.

Remission in UC is achieved with 5-aminosalicylic acid (5-ASA), glucocorticoids or biologics and disease severity influences the treatment modality. 5-ASA is the first-line choice to induce remission in mild to moderate UC patients, while glucocorticoid is reserved for patients with failure of response or those who are intolerant to 5-ASA because of the potential adverse events [5]. The serious side effects include osteoporosis and fracture, hyperglycemia, hypertension, gastric ulcer, mood disorders and necrosis of femoral head [6–8]. The increased susceptibility to infections, and with it an associated increase in mortality, was also seen in inflammatory bowel disease (IBD) patients on glucocorticoid therapy [5]. Although the emergence of biologics seems benefit the patients without response to these conventional therapy strategies, the high expense limited its use, especially in some developing countries where the health insurance does not cover. Thus, the introduction of therapy with high efficacy and safety in the early stage of disease course is necessary.

Fecal microbiota transplantation (FMT), as a whole set of healthy flora transplanted into patients, has been introduced as a treatment for UC. It modifies the gut microbiota with more diverse and more similar to those of the donors.Moayyedi et al. conducted a RCT involving the patients underwent FMT or placebo (water) retention enema. FMT was found to induce higher remission of UC in the FMT group than that in the placebo group (*p* = 0.03) [9]. Rossen et al. reported in their RCT with UC patients randomized to receive either donor FMT (FMT-D) or autologous FMT (FMT-A). Although the proportion of patients who achieved remission was not significantly different in FMT-D and FMT-A group, the microbiota of responders had distinct features from that of non-responders [10]. Costello et al. also enrolled UC patients into the FMT-D and FMT-A stool group, while they found FMT-D achieved favorable steroid-free remission over FMT-A (*p* = 0.03) [11]. Another RCT conducted by Paramsothy et al. assigned UC patients randomly to receive FMT versus placebo (isotonic saline). More patients in FMT group achieved the steroid-free remission than those in placebo group (*p* = 0.021) [12]. To date, all these RCTs included UC patients with mild to moderate severity to assess the efficacy of FMT with placebo or autologous FMT and the corresponding microbiota changes, however, no comparison of FMT with the conventional therapy has been conducted.

Since glucocorticoids still play an important role in the active UC, we intended to find out whether FMT could be applied as an alternative or supplement to this conventional treatment. Active mild to moderate UC patients who failed to reach clinical remission with 5-ASA monotherapy were enrolled into this prospective cohort and treated with either FMT or glucocorticoids. The primary objective was to assess and compare the efficacy and adverse events of FMT with glucocorticoids therapy. UC patients may benefit from the study for the treatment choice.to induce remission.

## Materials and methods

### Study design

This open prospective single-center cohort study was conducted in Shanghai General Hospital in China. We considered for inclusion of steroid-naïve UC patients with mild to moderate severity from September 2015 to September 2021. The study was approved by the ethics committee of the Shanghai General Hospital. Written informed consent was obtained from all the patients. This study was registered with ClinicalTrials.gov (NCT02435160).

### Study population

The diagnosis of UC was based on the guidelines of the European Crohn’s and Colitis Organizations [3]. Eligible patients were aged between 18 and 75 years and had a confirmed UC diagnosis for at least three months. Active UC with mild to moderate severity was defined as a total Mayo score between 4 and 10 with an endoscopic subscore ≥ 1 point. Those not reaching clinical remission with 5-ASA monotherapy after at least 8 weeks, were enrolled. Clinical remission was defined as no rectal bleeding and normal stool frequency (≤ 3/day) [3]. Patients previously exposed to topical mesalamine or glucocorticoids had a 2-week washout period before being enrolled. Patients receiving oral glucocorticoids and/or immunosuppressive agent and/or biologics treatment were excluded. Patients could not cooperate to complete the trial or unwilling to sign informed consent were not eligible for the study. Coexisting infections with enteric pathogens such as *Salmonella*, *Shigella*, *Escherichia coli O157 H7* and cytomegalovirus were screened before enrollment. Women who were pregnant were excluded. We also excluded those patients considered unfit for FMT to avoid selection bias.

### Donors and FMT solution preparation

The donors were screened from healthy volunteers between 18 and 50 years of age. They were assessed by a screening questionnaire. The exclusion criteria including: history of autoimmune diseases; history of gastrointestinal cancer, chronic constipation, irritable bowel syndrome and other digestive diseases; history of digestive system surgery; history of metabolic diseases such as diabetes and metabolic syndrome; receiving antibiotics within 3 months. The donor had to have negative serology for human immunodeficiency virus, hepatitis B, hepatitis C and syphilis. Urea breath test (UBT) was performed to exclude the potentially asymptomatic *H. pylori* carriers. The donor stool was further screened for enteric pathogens such as *Salmonella*, *Shigella*, *Yersinia*, *Campylobacter* and *Escherichia coli O157 H7*, as well as parasites and *Clostridioides difficile*. The donor stool was collected and purified within 2 h. 100 g stool was mixed with 500 ml of normal saline, and then vortexed for 10 min. The mixture was filtered twice with sterile gauze to remove the impurities, and finally poured into a 50 ml sterile container for storage at − 80 °C.

### Intervention and follow-up

All the participants were informed by the researchers of the potential advantages and disadvantages of FMT and GCs. It was left to the discretion of the patients which intervention of treatment they preferred.

The FMT administrations were performed in three consecutive days in FMT group. Each time 150 ml of purified FMT solution was administrated. The 1^st^ FMT administration was performed by a non-study endoscopist through endoscopic spray after oral administration of standard dose (2L) of compound polyethylene glycol electrolyte solution for intestinal preparation. Retention enema of FMT was performed on the following 2nd and 3rd days without additional laxative.

The patients in the glucocorticoids (GCs) group received oral prednisone 0.8–1 mg/kg/day. The prednisone dose was stable for 2 weeks, and then tapered by 5 mg per week. After the dose reaching 20 mg/day, a taper of 2.5 mg per week was performed.

Both groups received the original dose of oral 5-ASA treatment at a stable dose. Prohibited concomitant therapies included antibiotics and probiotics.

**Fig. 1:**
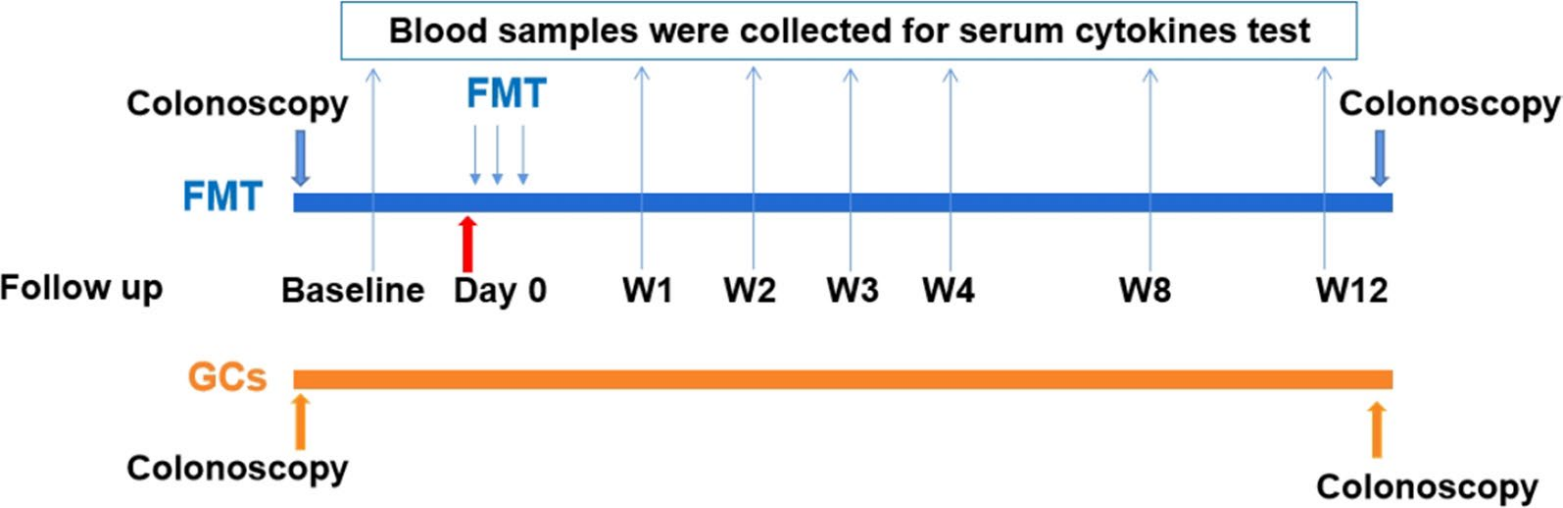
The participant timeline of the cohort. *FMT* fecal microbiota transplantation, *GCs* glucocorticoids

The patients were followed up at 1, 2, 3, 4, 8 and 12 weeks. The final follow-up was at week 12. The symptoms and signs of the patients were recorded, including fever, bloody stool, diarrhea and abdominal pain and distension. Blood sample was collected at each visit. Biochemical parameters and blood count were analyzed in the routine laboratory. The remaining blood was centrifuged at 6 °C and stored at – 80 °C within 30 min after withdrawal. The colonoscopy was performed at baseline and week 12 and the intestinal mucosa and the endoscopic Mayo score was assessed by two independent endoscopists who were blind to the intervention of the patients by examining photos and videos provided by the researchers. If the scores were different, another senior endoscopist would be invited to provide a final one based on the mucosal appearance and the former scores. The total Mayo score was determined at both baseline and week 12. Furthermore, the score on the Inflammatory Bowel Disease Questionnaire (IBDQ) was determined to assess the quality of life at baseline and week 12. Adverse events were recorded all through the study. The participant timeline concerning enrollment, intervention, assessment and visits for participants was shown in Fig. 1.

Patients who met the criteria for treatment failure or had a relapse and received rescue therapy, including intravenous steroid, biological therapy or surgery, were withdrawn. A relapse was defined as an increase in UC-related symptoms requiring consultation with a physician and leading to an increase in the dose of ongoing medical treatment, the introduction of new medication, or surgery [13].

### Sample size assumption

Based on an FMT response of 40% as reported by Rossen et al. [10] and an assumed glucocorticoids treatment effect of 65% according to integrated analysis of the data in different trials [14–16], a desired power of 80% and a 2-sided ɑ-level of 0.05, a total sample size of 120 was calculated for the primary outcome analysis.

### Outcomes

#### Primary outcome

Primary outcome was the reduction of the total Mayo score at week 12. The clinical and endoscopic remission was defined as a total Mayo score ≤ 2 points, with all Mayo subscores ≤ 1 point and improvement of endoscopic subscope ≥ 1 point compared with the baseline.

#### Secondary outcome

Key secondary outcomes were defined as following: clinical remission was defined as combined Mayo subscores of 1 or less for rectal bleeding plus stool frequency; clinical response as either a decrease of 3 points or more on the Mayo score; endoscopic remission as a Mayo endoscopic subscore of 0; endoscopic response as a Mayo endoscopic subscore of 1 or less. Adverse events were assessed by patient survey during follow-up. The quality of life was assessed by the scores of IBDQ.

Changes of inflammatory parameters [white blood cell (WBC) count, platelet count, C-reactive protein (CRP) and erythrocyte sedimentation rate (ESR)], hemoglobin, and albumin following FMT or GCs intervention was assessed at baseline and week 12. The biochemical parameters were measured using reagents by Beckman, according to the specification of the manufacturers.

Changes of serum cytokines (TNF-α, IFN-γ, IL-1β, IL-4, IL-5, IL-6, IL-8, IL-10, IL-12p70, IL-13, IL-17A and IL-23) were evaluated in FMT group at baseline and week 1, 2, 3, 4, 8 and 12. The participants following FMT were further defined as responders (RE) and non-responders (NR) according to the primary outcome. The results were then stratified to compare the levels of cytokines between RE and NR groups following FMT. The cytokines were measured in the multiplex bead assay using a Luminex200 system. A mixture of 50μL of 1:10 diluted antibody-immobilized fluorescent beads and 50 μL serum was incubated in 96-wells plate for 2 h at room temperature and continuously shaken. The plate was placed on the magnetic frame and washed for three times. Then 50 μL biotin labeled antibody complex was added, followed by incubation and shaking at room temperature for 1 h and washing for three times. 50 μL streptavidin labeled PE was added and incubated for 0.5 h. The washed samples were measured on Luminex200 flow analyzer.

### Statistical analysis

All variables of continuous data with normal distribution are expressed as mean (SD) and analyzed by *t*-test. The variables with non-normal distribution were expressed as median (IQR) and analyzed by Mann Whitney U-test. For categorical variables, data were expressed as numbers and percentages and evaluated by Chi-squared test. Differences with *p* values < 0.05 were considered significant.

## Results

### Characteristic of patients

From September 2015 to September 2021, 128 patients with mild to moderate UC (Mayo score 4–10) were recruited in Shanghai General Hospital. 6 patients were excluded for the following reasons: 1 for infection of cytomegalovirus; 3 for unqualified Mayo score; 1 for using antibiotics enema; 1 withdrew after screening.

**Fig. 2:**
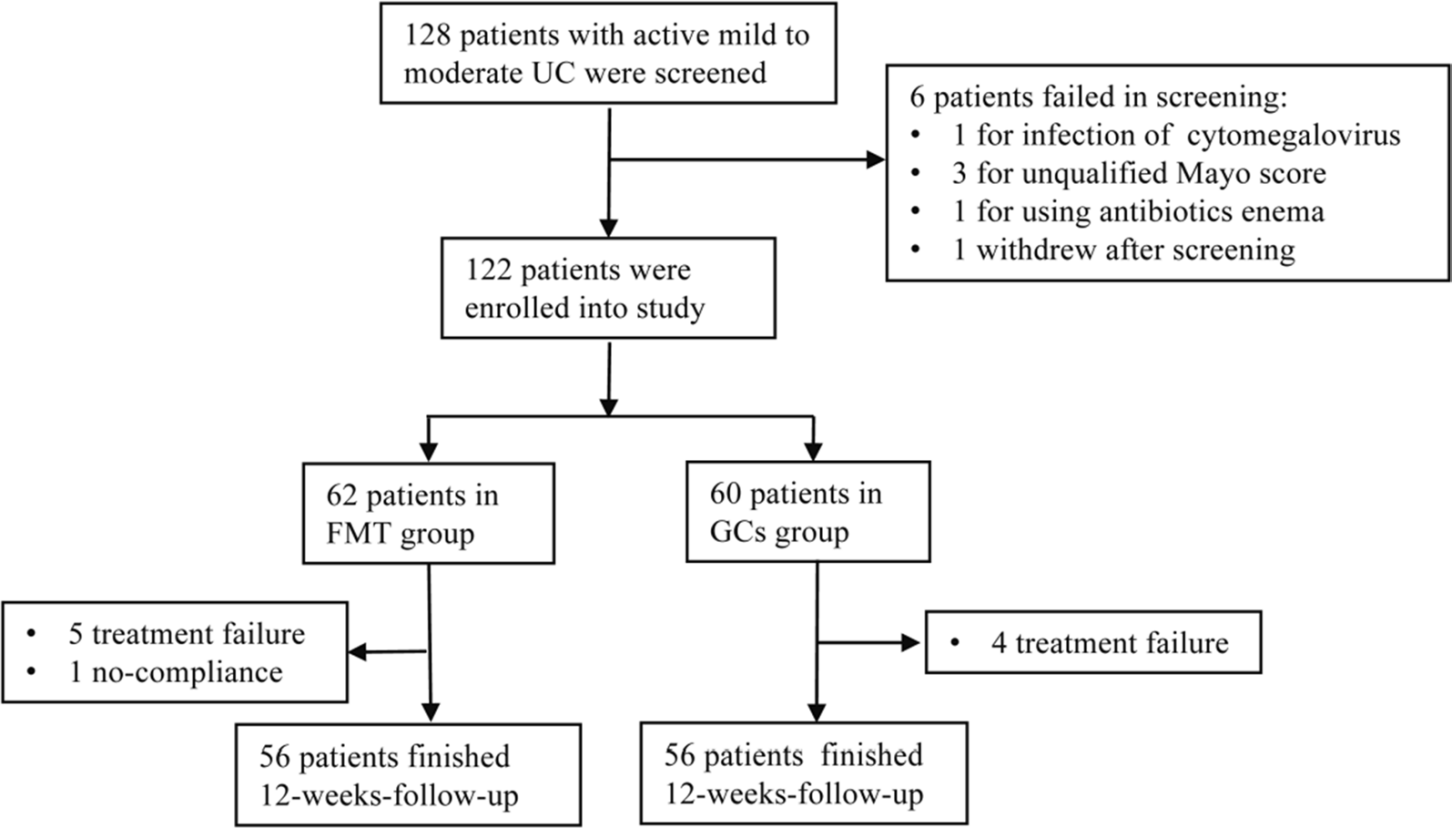
The flow of patients in the trial. *FMT* fecal microbiota transplantation, *GCs* glucocorticoids

Overall, 122 patients were considered for the trial, 62 were in FMT group and 60 in GCs group (Fig. 2). There was no statistically significant difference between the two groups at baseline concerning gender, age, disease duration, extent of the disease, disease severity (both the total Mayo scores and endoscopic Mayo scores) and the clinical inflammatory parameters (Table 1).

**Table 1:** Baseline characteristics of the study groups

Characteristics	FMT group (n = 62)	GCs group (n = 60)	*p* value
Gender, n (%)			
Male	44 (71.7)	43 (71.7)	0.93
Female	18 (28.3)	17 (28.3)	
Age, mean (SD)	43.1 (17.1)	47.4 (16.2)	0.16
Duration of disease, mean (SD)	2.7 (3.4)	3.4 (3.9)	0.33
Disease extent, n (%)			
E1, Proctitis	13 (21.0)	11 (18.3)	0.78
E2, Left-sided colitis	24 (38.7)	27 (45)	
E3, Pancolitis	25 (40.3)	22 (36.7)	
Concomitant medicine, n (%)			
Oral 5-ASA	62 (100)	60 (100)	
Topical 5-ASA	0	0	
Immunomodulator	0	0	
Biologics	0	0	
Total Mayo scores			
Mean (SD)	6.8 (1.9)	7.2 (2.0)	0.33
Range	(4–10)	(4–10)	
Endoscopic Mayo scores			
Mean (SD)	2.2 (0.6)	2.0 (0.6)	0.10
Range	(1–3)	(1–3)	
Inflammatory index			
WBC count, × 10^9^/L, mean (SD)	7.0 (2.4)	6.9 (2.5)	0.82
Neutrophils count, × 10^9^/L, mean (SD)	4.6 (2.2)	4.6 (2.0)	0.99
CRP, mg/L, median (IQR)	2.5 (0.7–7.9)	4.6 (0.8–12.8)	0.12
ESR, MM/H, median (IQR)	11 (5–25)	15 (5–37.3)	0.39
PLT count, × 10^9^/L, mean (SD)	256.8 (83.9)	250.5 (78.2)	0.66
Hemoglobin, g/L, mean (SD)	132.2 (19.5)	126.1 (23.4)	0.12
Albumin, g/L, mean (SD)	40.9 (5.2)	39.5 (5.6)	0.17

*FMT* fecal microbiota transplantation, *GCs* glucocorticoids, *5-ASA* 5-aminosalicylic acid

### FMT was as effective as glucocorticoids for UC remission induction

Six patients withdrew from the FMT group during the study therapy (Fig. 2). Three male patients with moderate UC in FMT group remained unwell and withdrew to receive intravenous steroid therapy. One male patient in FMT group needed antibiotics treatment at week 6 because of perianal abscess. One female patient in FMT group deteriorated at week 3 with large amount of bloody feces and needed hospitalisation for intravenous steroid and anti-tumour necrosis factor therapy. One male patient in FMT group withdrew because of non-compliance of using antibiotics during follow-up.

Four patients withdrew from the GCs study treatment (Fig. 2). One moderate female patient worsened during GCs treatment and refused to accept biologic therapy and underwent colectomy. One male patient following GCs withdrew because of clinical deterioration and CMV infection and underwent colectomy. One male patient in GCs group remained unwell and one male worsened during steroid reduction and then transferred to anti-tumour necrosis factor therapy.

34 cases (54.8%) in the FMT group and 29 (48.3%) in the GCs group reached the primary outcome, showing no significant difference in inducing clinical and endoscopic remission in UC (RR 1.13, 95% CI 0.8–1.6; *p* = 0.59) (Table 2). The proportions of patients following FMT therapy who reached the primary outcome were similar in early stage of UC disease (≤ 1 year) and chronic stage (> 1 year) (19/34, 55.9% *vs.* 15/28, 53.6%, *p* = 0.53).

**Table 2:** Primary and key secondary outcomes

	FMT group (n = 62)	GCs group (n = 60)	*p* value
Primary outcome, n (%)	34 (54.8)	29 (48.3)	0.59
Key secondary outcomes, n (%)			
Clinical remission	40 (64.5)	34 (56.7)	0.46
Clinical response	43 (69.4)	39 (65.0)	0.70
Endoscopic remission	19 (30.6)	17 (28.3)	0.84
Endoscopic response	38 (61.3)	35 (58.3)	0.85

*FMT* fecal microbiota transplantation, *GCs* glucocorticoids

40 participants in the FMT group (64.5%) and 34 in the GCs group (56.7%) had a clinical remission (RR 1.14, 95% CI 0.85–1.52; *p* = 0.46) at week 12. 43 participants in the FMT group (69.4%) and 39 in the GCs group (65.0%) had a clinical response (RR 1.14, 95% CI 0.83–1.37; *p* = 0.7). Both the endoscopic remission rate (19/62, 30.6% *vs.* 17/60, 28.3%) (RR 1.08, 95% CI 0.62–1.87; *p* = 0.84) and the endoscopic response rate (38/62, 61.3% *vs.* 35/60, 58.3%) (RR 1.05, 95% CI 0.79–1.40; *p* = 0.85) were a little higher in the FMT group than those in the GCs group, whereas no significant difference was found (Table 2).

As an open prospective cohort, administration of FMT or GCs was based on clinical practice and the discretion of the patients rather than randomization, which could have introduced bias into our analysis. Although baseline characteristic of the two groups showed no significant difference (Table 1), the subgroup analysis (Table 3) was further performed to avoid the potential selection bias. In the subgroup analyses according to age, gender, disease extent and duration of disease, no significant difference was found concerning the proportion of patients reaching primary outcomes between FMT and GCs groups.

**Table 3:** Subgroup analyses of primary outcomes

Subgroups, n (%)	FMT group (n = 62)	GCs group (n = 60)	*p* value
Age			
< 60	29 (59.2) (n = 49)	22 (52.4) (n = 42)	0.51
≥ 60	5 (38.5) (n = 13)	7 (38.9) (n = 18)	0.64
Gender			
Male	26 (59.1) (n = 44)	22 (51.2) (n = 43)	0.46
Female	8 (44.4) (n = 18)	7 (41.2) (n = 17)	0.56
Disease extent			
E1, Proctitis	9 (69.2) (n = 13)	7 (63.6) (n = 11)	0.56
E2, Left-sided colitis	13 (54.2) (n = 24)	14 (51.9) (n = 27)	0.87
E3, Pancolitis	12 (48.0) (n = 25)	8 (36.4) (n = 22)	0.42
Duration of disease, (years)			
≤ 1	19 (55.9) (n = 34)	12 (54.5) (n = 22)	0.92
> 1	15 (53.6) (n = 28)	17 (44.7) (n = 38)	0.48

*FMT* fecal microbiota transplantation, *GCs* glucocorticoids

**Fig. 3:**
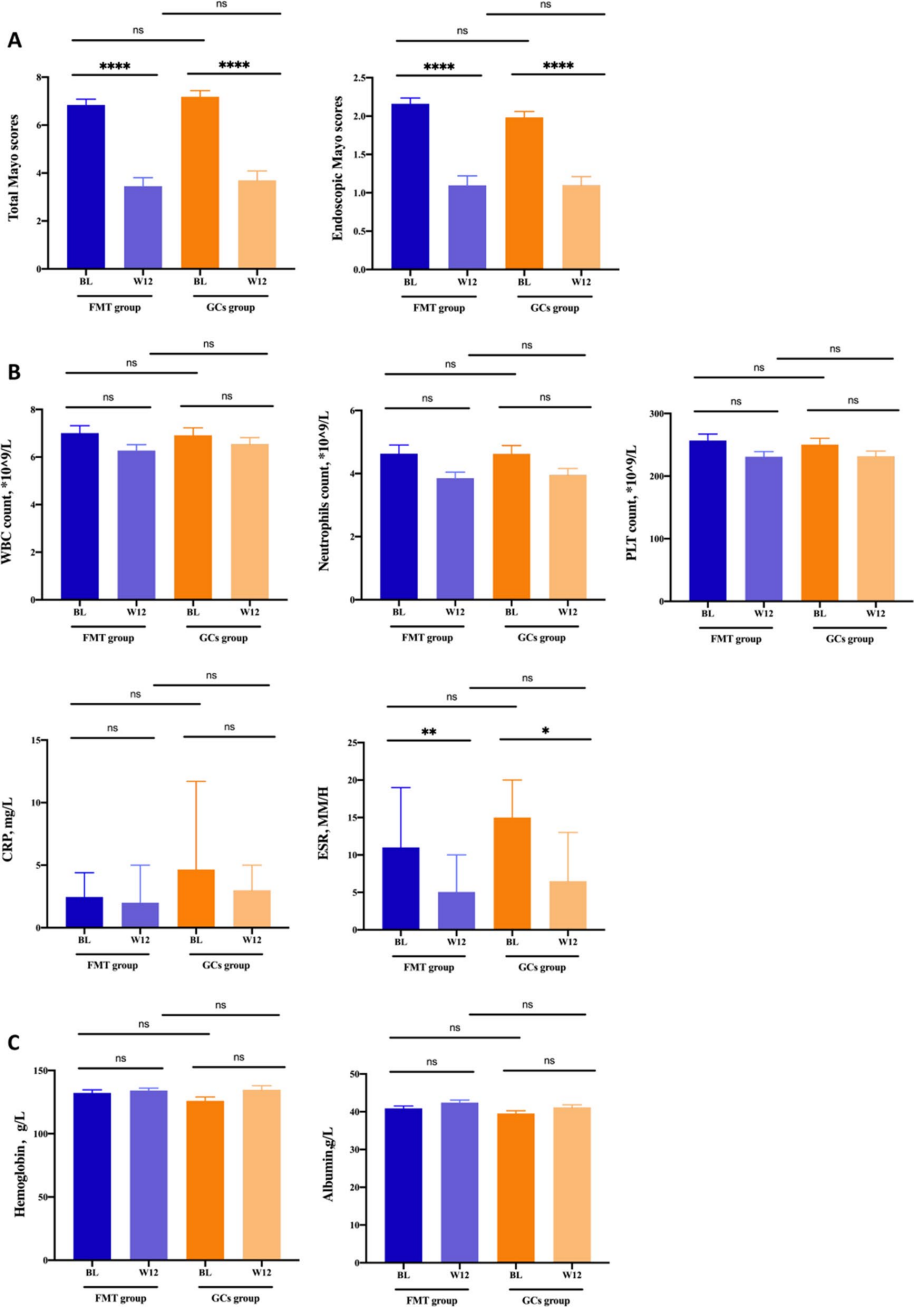
FMT was as effective as GCs for UC remission induction. **A** Total Mayo scores and Mayo endoscopic subscores. **B** Inflammatory index including WBC count, neutrophils count, PLT count and levels of CRP and ESR. C, Levels of Hemoglobin and albumin. *FMT* fecal microbiota transplantation, *GCs* glucocorticoids, *WBC* white blood cell, *CRP* C-reactive protein, *ESR* erythrocyte sedimentation rate, *PLT* platelet

The total Mayo score decreased significantly in both FMT and GCs groups after interventions (*p* < 0.001, respectively), while no significant difference was found between the two groups at week 12 (Fig. 3A). As far as Mayo endoscopic score was concerned, significant reduction were also found in two groups at week 12 compared with that at baseline (*p* < 0.001, respectively). The comparison of Mayo endoscopic score between two groups at week 12 showed no significant difference.

The levels of ESR decreased significantly in both FMT (*p* = 0.008) and GCs group (*p* = 0.023) following intervention, whereas decrease of other inflammatory parameters, including WBC count, neutrophils count, PLT count and level of CRP showed no significant difference (Fig. 3B). The level of serum albumin and hemoglobin increased in both groups at week 12, while no significant difference was found (Fig. 3C).

### Less adverse events were observed in FMT group

In FMT group, 14 patients (22.6%) had at least one adverse event reported, mostly mild and self-limiting gastrointestinal discomfort and fever. In GCs group, 36 patients (60.0%) had at least one adverse event reported, such as colectomy, leukopenia, abnormal liver function, electrolyte disorder, cytomegalovirus infection, hypertension, hyperglycemia, gastrointestinal discomfort, and insomnia. The adverse events in the GCs group were significantly higher than those in the FMT group (*p* < 0.001) (Table 4). Two serious adverse events were observed during the GCs therapy. One moderate female patient worsened during GCs treatment and refused to accept biologic therapy and underwent colectomy. One male patient showed deterioration of colitis and was then diagnosed with CMV infection and underwent colectomy.

**Table 4:** Adverse events in FMT and GCs groups

	FMT group (n = 62)	GCs group (n = 60)	*p* value
Patients with adverse events, n (%)	14 (22.6)	36 (60.0)	
Total events (n)	17	68	*p* < 0.001
Colectomy	0 (0)	2 (33.3)	
Leukopenia, n (%)	0 (0)	3 (5)	
Abnormal liver function, n (%)	0 (0)	2 (3.3)	
Electrolyte disorder, n (%)	0 (0)	10 (16.7)	
Cytomegalovirus infection, n (%)	0 (0)	2 (3.3)	
Hyperglycemia, n (%)	0 (0)	6 (10)	
Hypertension, n (%)	0 (0)	4 (6.7)	
Gastrointestinal discomfort, n (%)	12 (19.4)	18 (30.0)	
Fever, n (%)	5 (8.1)	0 (0)	
Insomnia, n (%)	0 (0)	21 (35)	

*FMT* fecal microbiota transplantation, *GCs* glucocorticoids

### Quality of life assessment showed improvement in both FMT and GCs groups

**Fig. 4:**
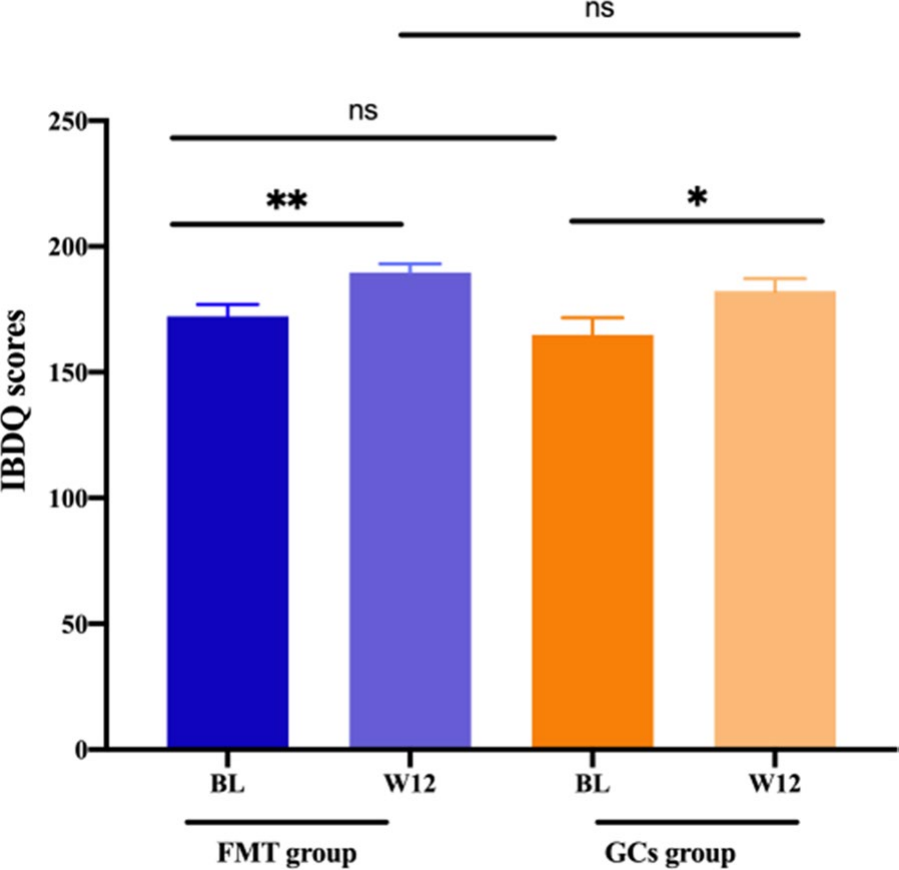
IBDQ score measured at baseline and week 12 in FMT and GCs groups. *FMT* fecal microbiota transplantation, *GCs* glucocorticoids, *BL* baseline, *IBDQ* Inflammatory Bowel Disease Questionnaire

The participants were asked to fill the IBDQ and 56 patients per group completed the IBDQ at baseline and week 12. The IBDQ score increased significantly in both FMT and GCs groups after intervention (Fig. 4). Although the scores in the GCs group were a little lower compared with those in the FMT group after intervention, no significant difference was found.

### Reduction of TNF-α and IL-6 level in FMT group was related with the remission

The serum cytokines including TNF-α, IFN-γ, IL-1β, IL-4, IL-5, IL-6, IL-8, IL-10, IL-12p70, IL-13, IL-17A and IL-23 were measured and analyzed in FMT group at baseline and 1, 2, 3, 4, 8 and 12 weeks after FMT intervention.

**Fig. 5:**
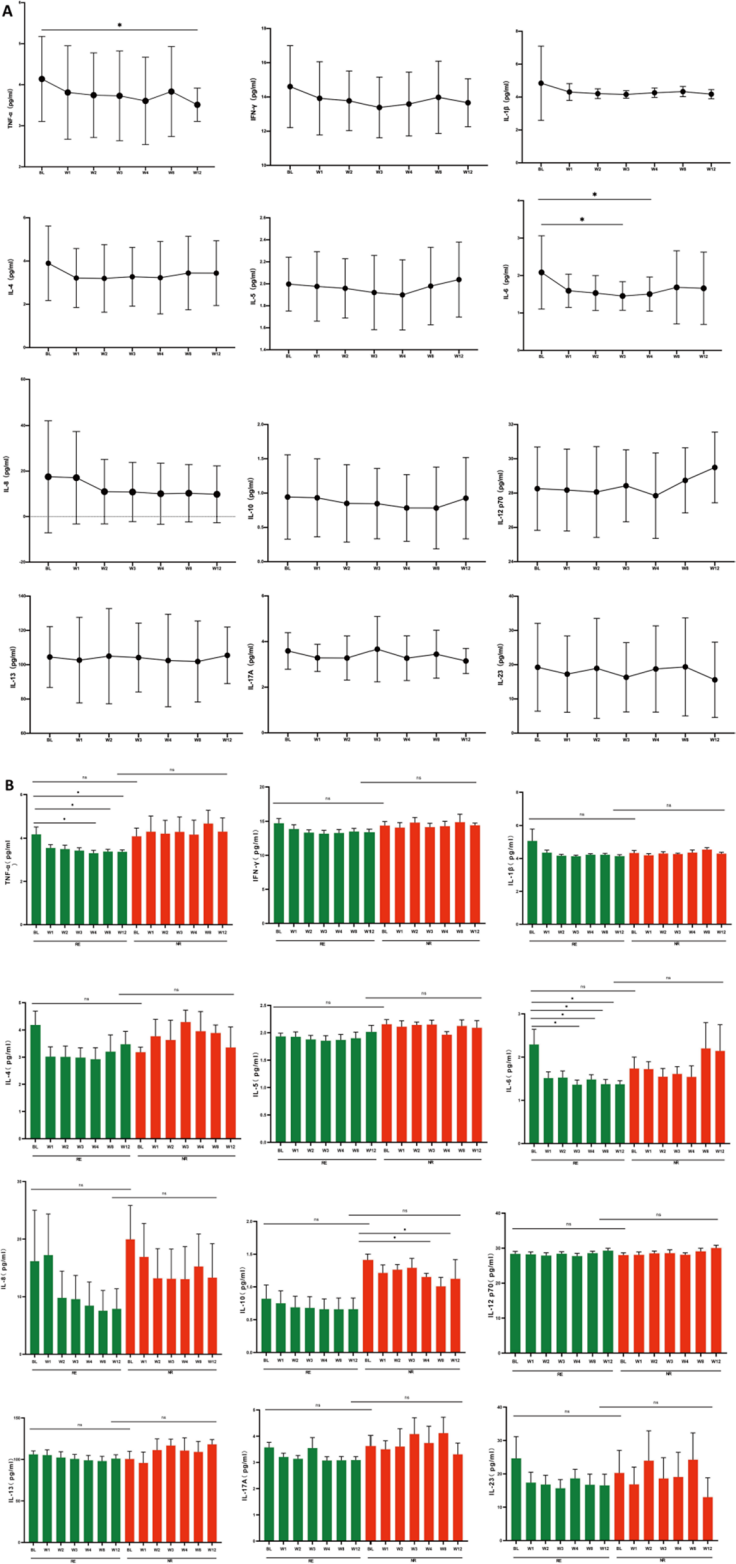
Reduction of TNF-ɑ and IL-6 level in FMT group was related with the remission in FMT group. **A** the inflammatory factors at baseline and after FMT intervention. **B** the comparison of inflammatory factors between RE and NR groups following FMT. *FMT* fecal microbiota transplantation, *GCs* glucocorticoids, *BL* baseline, *RE* responders, *NR* non-responders

The levels of TNF-α, IFN-γ, IL-1β, IL-6 and IL-8 showed obvious decreasing trend after FMT therapy (Fig. 5A). The level of TNF-α decreased significantly at week 12 compared with that at baseline, while IL-6 at week 3 and 4 decreased significantly compared with that at baseline, respectively (*p* < 0.05).

The 62 participants in FMT group were then classified into RE group (n = 34, 54.8%) and NR group (n = 28, 45.2%) according to the primary outcome at week 12. The serum cytokines were further analyzed between RE and NR groups before and after FMT intervention (Fig. 5B).

For all the cytokines no significant difference was found at baseline between RE and NR groups. Although descending trend was found in most of the cytokines in RE group, only TNF-α and IL-6 showed significant reduction after FMT therapy (*p* < 0.05). Compared with that at baseline, the level of TNF-α decreased significantly at week 4, 8 and 12, while IL-6 decreased significantly at week 3, 4, 8 and 12, respectively (*p* < 0.05). Whereas, no significant change of TNF-α or IL-6 was found in NR group. On the other side, the reduction trend of IL-10 was more obvious in NR group than that in RE group and significant decrease was shown at week 4 and 12 in NR group (*p* < 0.05).

Although none of the cytokines showed significant difference at week 12 after FMT intervention between RE and NR groups, the decrease of TNF-α and IL-6 was considered to be associated with remission in UC following FMT, while the decrease of IL-10 with non-remission.

## Discussion

This was the first cohort to compare FMT with GCs on inducing remission in UC with mild to moderate severity and showed similar effect of FMT to GCs. The proportion of patients with clinical and endoscopic improvement were not statistically significant between two therapy groups.

The usefulness of cortisone in UC was firstly established by the Medical Research Council trial in 1955 [14]. Oral prednisone was then presented and brought about rapid remission of the disease in a high proportion of cases [15]. It is stated in ACG guideline that in patients with UC of any extent who fail to respond to 5-ASA therapy, oral systemic corticosteroids should be recommended to induce remission [17]. Due to the potential adverse effects, second generation of GCs, such as budesonide, budesonide MMX, Beclomethasone dipropionate, were then taken as alternatives to conventional treatment [18]. However, budesonide was less likely to induce clinical remission than oral 5-ASA and MMX 9-mg budesonide preparation also failed to demonstrate a benefit over placebo in mild to moderate extensive UC [19]. Moreover, GCs resistance or refractoriness is still an unresolved issue for GCs treatment, which is the inability of GCs to exert their effects [20].

In our study, two patients in GCs group with deterioration of colitis and underwent colectomy. Some studies have focused on searching the effective therapy in steroid-refractory UC patients in avoiding a colectomy. A retrospective study indicated that both cyclosporine and infliximab were effective in avoiding a colectomy in steroid-refractory UC patients. During the follow-up the risk of a colectomy was higher in patients treated with cyclosporine than with infliximab [20]. Another retrospective analysis reported that initial treatment with a calcineurin inhibitor in combination with vedolizumab allowed more than two thirds of patients active steroid-refractory UC patients (most refractory to a tumor necrosis factor antagonist) to avoid colectomy [21]. In our study, two steroid-refractory patients transferred to anti-tumour necrosis factor therapy and had excellent prognosis.

One of the two male patients undergoing colectomy appeared to have the diagnosis of CMV infection. CMV infection and steroid use have been identified as risk factors for hospitalization because of UC aggravation [22]. The burden of CMV colonic reactivation in patients with active UC seems to be related to the risk of colectomy [23].

FMT has been used in various diseases related to gut microbiota, with the most promising outcome reported in *Clostridioides difficile* infection (CDI), followed by IBD. Intestinal dysbiosis has been long suspected to be a major risk factor in etiology of UC, however, the identification of such ‘dysbiotic’ microbiota in gut and underlying mechanisms in the host has been challenging. Several different RCTs have reported intestinal compositions changes after FMT in UC with great variation [9–12]. Although no more obvious efficiency of FMT was found in the UC patients in the early stage (≤ 1 year) than that in the chronic stage in our study, it has been reported that FMT may be more efficacious in patients with a recent diagnosis of UC, as a perturbation in the microbiome might be more easily restored early in the course of the disease [9].

Furthermore, whether the effect of FMT is attributable to changing certain metabolites has also been focused on. It has been reported in a RCT that there were increased levels of SCFA and secondary bile acids in patients who experience UC remission after FMT, while patients who did not response were observed to have enhanced heme and lipopolysaccharide biosynthesis [24].

The defects in microecology, with the related metabolic pathways and molecular mechanisms, play a critical role in the innate immunity of the intestinal mucosa in UC [25], which is strongly related to the pathogenesis of UC [26]. CD4^+^ T cells are the main effector lymphocyte cells which contribute to a dysregulation of the production of several cytokines leading to intestinal inflammation in UC, including Th2 signature cytokines IL-4, IL-5 and IL-13 and Th17 cells producing IL-17 [27]. Over-expression of IL-6, IL-8, IL-17 and TNF-ɑ in inflamed mucosa or serum is also reported to be related to disease severity in UC [28]. Colonization with IBD microbiota induced more Th17 cells and a population of highly pro-inflammatory T cells co-expressing the cytokines IL-17 and IFN-γ.[29] Regulation of these cytokines is generally mediated by the immunoregulatory cytokine, such as IL-10 or TGF-β produced by Tregs [30].

In our study, serum TNF-ɑ and IL-6 was decreased significantly in UC patients after FMT and further stratified analysis showed the decrease of TNF-ɑ and IL-6 was observed only in UC patients responding to FMT. IL-6 was reported to orchestrate a series of 'downstream' cytokine-dependent signaling pathways that amplify RORγt-dependent differentiation of Th17 cells [31]. With regard to IL-10, as anti-inflammatory cytokines, enhanced level was reported in some studies [29, 32, 33]. However, in our study, significant decrease of IL-10 was observed in non-responders following FMT, while no obvious change was found in responders, probably presenting IL-10 to be essential for the anti-inflammatory properties in UC. These results indicated that FMT might have an anti-inflammatory effect to induce remission in UC which may result from the modulation of gut microbiota related cytokine expression.

This study had some limitations. Although it was observed in our cohort that FMT might be as effective as GCs in active UC patients and could be used as a feasible alternative to conventional treatment with fewer adverse events, the underlying mechanism still needs to be studied. We explored the serum cytokines changes between the responders and the non-responders with FMT intervention, however, the potentially related gut microbiota modulation had not been studied. Further study concerning the relationship between changes in intestinal immunological function and microbiota structure should be focused on, which is suggestive of a causative mechanism for UC remission.

## Conclusions

We compared the clinical efficacy and safety of FMT with GCs on active mild to moderate UC patients, together with changes in systemic inflammatory conditions. FMT was as effective as GCs to be a remission induction therapy with fewer adverse events. Therefore, FMT might be more suitable for UC patients with mild to moderate severity in the early phrase without repeated relapses and also substitute for GCs therapy in some patients with misgiving or intolerance to GCs treatment well. The efficacy of FMT for treating UC might be related with the modification of key cytokines such as TNF-ɑ, IL-6 and IL-10.

## Data Availability

Data are available from the authors upon request.

## Abbreviations

FMT: Fecal microbiota transplantation
UC: Ulcerative colitis
GCs: Glucocorticoids
RE: Responder
NR: Non-responders
5-ASA: 5-Aminosalicylic acid
IBD: Inflammatory bowel disease
FMT-D: Donor FMT
FMT-A: Autologous FMT
UBT: Urea breath test
WBC: White blood cell
CRP: C-reactive protein
ESR: Erythrocyte sedimentation rate
CDI: *Clostridioides difficile* Infection
IBDQ: Inflammatory Bowel Disease Questionnaire
